# Neonatal near-misses in Ghana: a prospective, observational, multi-center study

**DOI:** 10.1186/s12887-019-1883-y

**Published:** 2019-12-23

**Authors:** Ashura Bakari, April J. Bell, Samuel A. Oppong, Yemah Bockarie, Priscilla Wobil, Gyikua Plange-Rhule, Bamenla Q. Goka, Cyril M. Engmann, Richard M. Adanu, Cheryl A. Moyer

**Affiliations:** 1 Department of Child Health, Suntreso Government Hospital, Ghana Health Service, Kumasi, Ghana; 2 Global REACH, University of Michigan Medical School, Ann Arbor, MI USA; 3 Department of Obstetrics and Gynecology, Medical School, University of Ghana, Accra, Ghana; 4 Department of Child Health, Cape Coast Teaching Hospital, Cape Coast, Ghana; 5 Department of Child Health, Komfo Anokye Teaching Hospital / Kwame Nkrumah University of Science and Technology, Kumasi, Ghana; 6 Department of Child Health, Medical School, University of Ghana, Accra, Ghana; 7 Departments of Pediatrics and Global Health, University of Washington Schools of Medicine and Public Health, Seattle, WA USA; 8 Maternal, Newborn Childhealth and Nutrition, PATH, Seattle, WA USA; 9 Population, Family and Reproductive Health Department, University of Ghana School of Public Health, Accra, Ghana; 10 Departments of Learning Health Sciences and Obstetrics & Gynecology, University of Michigan Medical School, 1111 E. Catherine Street, 231 Victor Vaughan Bldg, Ann Arbor, MI 48109 USA

**Keywords:** Neonatal morbidity, Neonatal mortality, Neonatal near-miss indicators

## Abstract

**Background:**

For every newborn who dies within the first month, as many as eight more suffer life-threatening complications but survive (termed ‘neonatal near-misses’ (NNM)). However, there is no universally agreed-upon definition or assessment tool for NNM. This study sought to describe the development of the Neonatal Near-Miss Assessment Tool (NNMAT) for low-resource settings, as well as findings when implemented in Ghana.

**Methods:**

This prospective, observational study was conducted at two tertiary care hospitals in southern Ghana from April – July 2015. Newborns with evidence of complications and those admitted to the NICUs were screened for inclusion using the NNMAT. Incidence of suspected NNM at enrollment and confirmed near-miss (surviving to 28 days) was determined and compared against institutional neonatal mortality rates. Suspected NNM cases were compared with newborns not classified as a suspected near-miss, and all were followed to 28 days to determine odds of survival. Confirmed near-misses were those identified as suspected near-misses at enrollment who survived to 28 days. The main outcome measures were incidence of NNM, NNM:mortality ratio, and factors associated with NNM classification.

**Results:**

Out of 394 newborns with complications, 341 (86.5%) were initially classified as suspected near-misses at enrollment using the NNMAT, with 53 (13.4%) being classified as a non-near-miss. At 28-day follow-up, 68 (17%) had died, 52 (13%) were classified as a non-near-miss, and 274 were considered confirmed near-misses. Those newborns with complications who were classified as suspected near-misses using the NNMAT at enrollment had 12 times the odds of dying before 28 days than those classified as non-near-misses. While most confirmed near-misses qualified as NNM via intervention-based criteria, nearly two-thirds qualified based on two or more of the four NNMAT categories. When disaggregated, the most predictive elements of the NNMAT were gestational age < 33 weeks, neurologic dysfunction, respiratory dysfunction, and hemoglobin < 10 gd/dl. The ratio of near-misses to deaths was 0.55: 1, yet this varied across the study sites.

**Conclusions:**

This research suggests that the NNMAT is an effective tool for assessing neonatal near-misses in low-resource settings. We believe this approach has significant systems-level, continuous quality improvement, clinical and policy-level implications.

## Background

In 2019, 2.5 million newborns globally died within a month of birth, with nearly 2 million dying in the first week after birth [1]. While these figures represent significant improvements over previous decades, for every newborn who dies, as many as eight more suffer life-threatening complications but survive [2–4]. Such events, termed ‘near-misses,’ are becoming increasingly important indicators for not only epidemiologic surveillance but also assessment of quality of care. While there is a widely accepted World Health Organization maternal near-miss tool [5], there is much less clarity about the assessment of neonatal near-misses.

Several scoring tools have been used to assess severe morbidity among neonates, and to date, no standard criteria exist for neonatal near-misses [6, 7]. Neonatal near-misses have been defined as newborns who suffer a life-threatening condition following birth and survive the first 28 days of life [6, 7], but other definitions have utilized a shorter time-span of 7 days [8]. There is also disagreement on the most appropriate markers of illness severity indicative of a near-miss. Researchers from Brazil have used a variety of criteria to categorize a newborn as a near-miss, including “pragmatic markers” with various cut points, such as low birthweight (< 1500 g or < 1750 g), low gestational age at birth (less than 30, 31,32, or 33 weeks), or a five-minute Apgar below 5 or 7 [3, 5, 6, 8]. Some have added the use of mechanical ventilation or congenital malformation to the pragmatic criteria [5] ,while others have added management criteria [8, 9]. By contrast researchers utilizing data from Morocco, Burkina Faso and Uganda have used clinical features, the presence of organ-system dysfunction, and management criteria to classify a newborn as a near-miss, similar to the WHO maternal near-miss framework [10, 11]. This study was conducted in response to the criticism that the previous near-miss assessment tools relied heavily on technology that was not available in many low-resource settings, hence limiting their utility.

This study aimed to: 1) **describe the development of the Neonatal Near Miss Assessment Tool (NNMAT)** for low-resource settings; 2) identify the **incidence of neonatal near-misses** at two tertiary care hospitals in southern Ghana; 3) **compare the incidence rates of neonatal near-misses to institutional records of neonatal mortality (mortality that occurred within the hospital)**; and 4) to identify the **strongest predictors of death** when comparing newborns who experienced a neonatal near-miss with newborns known to have died within the first 28 days after birth.

## Methods

This was a prospective, observational, multi-site study conducted April–July 2015 at two tertiary referral hospitals in southern Ghana [12]. Globally accepted STROBE guidelines were used in designing and reporting findings [13].

### Setting

Study sites included the maternal and neonatal units of Komfo Anokye Teaching hospital (KATH) in Kumasi and Cape Coast Teaching Hospital (CCTH) in Cape Coast. KATH is the teaching hospital affiliated with the Kwame Nkrumah University of Science and Technology School of Medical Sciences (KNUST-SMS) and serves as the referral center for most of central Ghana. Each year approximately 11,000 women give birth at KATH. CCTH is the teaching hospital of the University of Cape Coast, School of Medical Sciences (UCC-SMS) and serves as the main referral hospital for much of the rural central and parts of western regions of Ghana. The hospital oversees about 2800 births per year. At the time of this research, each hospital provided specialty care for sick newborns, with capacity to provide bag and mask ventilation, oxygen and incubator care with radiant warmers (although often shared), and phototherapy. Routine laboratory tests, such as complete blood counts (CBC), serum bilirubin and electrolytes, were available, but delays in processing sometimes limited the utility and utilization of such tests.

### Instruments used

Given the lack of a universally accepted neonatal near-miss screening tool, [3, 6, 7] we reviewed the existing literature for research assessing neonatal near-misses [4–8, 14, 15] and examined the framework of the WHO Maternal Near-Miss Screening Tool [16]. We also reviewed modifications of the WHO Maternal Near-Miss Screening Tool for use in Ghana [17]. Neonatal tools to date have focused on “pragmatic markers of severity” (e.g. Apgar scores < 7 at 5th minute, birthweight < 1750 g, gestational age < 33 weeks) and “management markers of severity” (e.g. use of IV antibiotics, nasal CPAP, any intubation in the first 7 days, use of phototherapy, cardiopulmonary resuscitation, etc.), [3, 4, 15] while the WHO Maternal Near-Miss Tool focuses on symptoms, interventions, and organ system dysfunction to categorize mothers as having experienced a maternal near-miss. We consolidated all criteria found in the literature across the neonatal tools, and then used an interactive heuristic approach consisting of structured interviews with the heads of the departments of pediatrics and the supervisors of the NICUs at KATH, CCTH, and Korle Bu Teaching Hospital in Accra (affliated with the University of Ghana) and an international neonatalogist to determine the appropriateness of the included criteria for the Ghanaian setting. Each criterion was discussed at length, and cut points were agreed upon as most appropriate in this setting. The final tool, termed the **NNMAT** (Neonatal Near Miss Assessment Tool), was iteratively reviewed and revised, resulting in a tool with four categories: evidence of severe complications (akin to the “pragmatic category” seen in the literature), interventions conducted (akin to “management markers of severity”), organ-based dysfunction, and investigations conducted. Definitions conformed to the International Statistical Classification of Diseases and Health Related Problems (ICD-10). (See Table 1.)

**Table 1:** Neonatal Near-Miss Assessment Tool (NNMAT) Items^a^

**Category 1: EVIDENCE OF SEVERE/LIFE THREATENING COMPLICATIONS**	**Category 2: CLINICAL INTERVENTIONS SUGGESTIVE OF A NEAR-MISS**
Apgar Score < 7 at 5 min	Resuscitation (bag & mask) in the first minute
Gestational Age < 33 weeks	Resuscitation in the NICU (after the first minute)
Birthweight < 1800 g	Nasal CPAP
Suspected subgaleal bleed	Cardiac massage/Chest compressions
Major congenital abnormality requiring surgical repair (e.g. gastroschisis, hydrocephalus, duodenal artresia, congenital heart defect)	IV fluid bolus
Axillary temperature < 35 or > 39	Any intubation during admission
Severe jaundice requiring blood exchange	Double phototherapy
Surgery in first week	Double blood exchange transfusion
	Oxygen therapy
	IV fluid 12h hours
	Caffeine citrate/aminophylline therapy
	Thermal protection > 4 h
**Category 3: ORGAN DYSFUNCTION**	**Category 4: INVESTIGATIONS IN THE FIRST 7 DAYS**
Cardiovascular:	Haematocrit < 30%
Capillary refill time > 3 s	Hemoglobin < 10gd/dl
Persistent tachycardia > 180 bpm	White blood cells < 4000 cells/mm3
Persistent bradycardia < 80 bpm	Serum bilirubin level > 10 x gestational age
Cardiac arrest	Blood culture done
Neurologic:	Blood culture positive
Recurrent seizures	
Abnormal posturing	
Inability to suck	
Floppy	
Poor Feeding	
Weak Cry	
Respiratory:	
Tachypnea > 100 cpm	
Bradypnoea < 20 cpm	
Grunting	
Cyanosis in air	
Gasping	
Chest in drawing	
Apnea	
Renal:	
Oliguria or anuria > 24 h	
Gastrointestinal:	
Persistent vomiting	
Distended abdomen	

^a^All assessed as yes/no/don’t know, with a yes in any category rolling up to a classification as a suspected ‘near-miss’, which was confirmed with survival to 28 days

### Study participants

All babies born at KATH and CCTH with birth complications indicated in the birth registry during the study period (e.g. pre-term birth, low birthweight (< 2500 g), Apgar scores < 7 at 5 min, birth asphyxia, congenital malformations, or indications that they were being transferred to the NICU) were identified and screened with the Neonatal Near Miss Assessment Tool (NNMAT) to identify potential near-miss cases. In addition, all babies admitted to the neonatal units at KATH and CCTH – regardless of where they were born – were recruited for participation as soon as possible following admission to the neonatal units. Babies less than 28 weeks gestation (the official age of viability in Ghana, estimated via date of last menstrual period or ultrasound scan if available) or less than 500 g birthweight were excluded, as were those who were stillborn.

### Data collection

Trained research assistants reviewed the admission ledgers at each site to identify newborns with complications. Parents of newborns with complications who had been admitted were approached, asked about their willingness to participate in a study and were taken through a written consent process if they agreed. The admission ledger was used to complete the NNMAT, supplementing information from the medical record with information from the physician or nurse on duty as necessary. NNMAT forms were completed using Qualtrics data collection software (Provo, Utah) on a hand-held tablet as soon as possible after admission, typically within 24 h. Babies were followed at 7, 14, and 28 days to determine survival. For those discharged home, families were contacted via telephone and asked to provide an update on the health of the baby.

### Key variables

In addition to generating the NNMAT, the two primary outcome variables of interest in this study were near-miss categorization and death. Table 1 illustrates the components of the NNMAT. Each item was recorded as yes, no, or don’t know. A positive response to an individual item yielded a positive classification within its overarching category, and any positive classification to any category was considered to be a neonatal near-miss. Thus newborns who had positive answers to any of the screening criteria were considered to be a suspected neonatal near-miss at enrollment, and a confirmed near miss if they survived to 28 days in keeping with the definition used by Silva et al. [5]. Death was assessed in two ways. First, aggregate, hospital-level statistics regarding institutional neonatal mortality were retrieved for the months of the study to be able to compare rates of near-misses with rates of reported institutional neonatal mortality. In addition, research assistants at each site continued to track every newborn recruited into the study for 7, 14, and 28 days after birth to determine survival. Those who died prior to 28 days were considered neonatal deaths for the purposes of identifying predictors of death vs. survival among those categorized as near-misses.

### Data analysis

Data were exported from Qualtrics into Stata version 13.1 (College Station, Texas) for cleaning and analysis. Frequencies were generated across all indicators assessed. Summary statistics reflecting total number of births, live births, and neonatal deaths for the study period were collected from each hospitals’ records, allowing for the calculation of neonatal near-miss incidence. Neonatal near-miss incidence was calculated by dividing the number of neonatal near-misses recorded by the number of live births recorded, in keeping with the predominant method of the existing literature [18]. Confidence intervals were calculated.

Pearson’s Chi Square analysis was conducted to compare those who died with those who survived the first 28 days after birth. Multivariate logistic regression was conducted (with death before 28 days as the outcome variable) to determine which categories of the neonatal near-miss tool were most predictive and then which elements within each category were most predictive. Variables significant at *p* < .05 in these preliminary models were carried forward into a final multivariate model to illustrate the strongest predictors of mortality. For the final model, a conservative *p* value of 0.01 was taken as statistically significant to account for multiple comparisons. In addition, sensitivity, specificity, positive predictive values, and negative predictive values of the NNMAT were calculated.

### Ethical review

Ethical approval was obtained from the Institutional Review Boards of Kwame Nkrumah University of Science and Technology, University of Cape Coast, University of Ghana, and the University of Michigan.

## Results

A total of 394 newborns across the two sites were enrolled, screened with the NNMAT, and successfully followed to 28 days. A total of 441 newborns were initially enrolled, yet 47 babies were lost to follow-up. Those 47 babies were less likely to be suspected near-misses at enrollment than those successfully followed to 28 days (*p* <  0.001), yet there was no difference in terms of location of birth, vaginal birth vs. c-section, or study site between those lost to follow-up and those successfully followed. Each site had an 89% retention rate.

Table 1 illustrates the criteria used to determine whether a newborn was classified as a suspected near-miss, and Table 2 illustrates the indicators that were present upon admission for all babies, those babies not classified as a near-miss, those who ultimately survived the first month (confirmed near-misses), and for those who died within 28 days.

**Table 2:** Criterion-based Causes of Neonatal Near-Misses in Southern Ghana

	All babies at enrollment (*N* = 394) N(%)	Indicators at 28-day follow-up			
		Not classified as a near-miss (*N* = 52) N(%)	Confirmed near-misses at 28 days (*N* = 274) N(%)	Babies who died within 28 days of birth (*N* = 68) N(%)	Chi^2^ *P* Value
NNMAT Category 1: Evidence of severe complication (yes to any of the following: Apgar score < 7 at 5 min (*n* = 193), gestational age < 33 weeks (*n* = 150), birthweight < 1800 g (*n* = 196), suspected subgaleal bleed (*n* = 39), major congenital abnormality (*n* = 37), axillary temperature < 35 °C or > 39 °C (*n* = 131), severe jaundice that required blood exchange (*n* = 15), surgery in first week (*n* = 4))	260 (66.0)	0 (0)	204 (74.5)	56 (82.4)	< 0.001
NNMAT Category 2: Intervention-Based Criteria (yes to any of the following: Resuscitation (bag & mask) immediately after birth (*n* = 201), resuscitation in the NICU (*n* = 208), chest compressions/cardiac massage (*n* = 15), IV fluid bolus (*n* = 178), intubation (*n* = 11), double phototherapy (*n* = 64), double volume exchange blood transfusion (*n* = 19), oxygen therapy (*n* = 323), caffeine citrate/aminophylline (*n* = 169), thermal protection (*n* = 188))	293 (74.4)	0 (0)	233 (85.0)	60 (88.2)	< 0.001
NNMAT Category 3: Organ-Dysfunction-Based Criteria (yes to specific indicators for cardiovascular (*n* = 31), respiratory (*n* = 148), renal (*n* = 56), gastroenterologic (*n* = 17), neurologic (*n* = 292))	186 (47.2)	0 (0)	134 (48.9)	52 (76.5)	< 0.001
NNMAT Category 4: Investigation-based criteria (yes to any: haematocrit > 30% (*n* = 8), hemoglobin < 10gd/dl (*n* = 10), serum bilirubin >10x gestational age (*n* = 9), positive blood culture (*n* = 7))	23 (5.8)	0 (0)	16 (5.8)	7 (10.3)	0.058
Met no criteria	53 (13.5)	52 (100)	0 (0)	1 (1.5)	< 0.001
Met one category of criteria	76 (19.3)	0 (0)	69 (25.2)	7 (10.3)	
Met two categories of criteria	120 (30.5)	0 (0)	106 (38.7)	14 (20.6)	
Met three categories of criteria	134 (34.0)	0 (0)	90 (32.9)	44 (64.7)	
Met four categories of criteria	11 (2.8)	0 (0)	9 (3.3)	2 (2.9)	

Out of all newborns followed, 66% (*N* = 260) exhibited some evidence of severe complications at enrollment, and 74.4% (*N* = 293) underwent interventions indicative of near-miss status within 24 h after birth. Slightly less than half (*N* = 186) qualified as a suspected near-miss based upon organ dysfunction criteria. Only 5.8% (*N* = 23) qualified based upon investigation-based criteria. A total of 341 were classified as a suspected neonatal near-miss, based upon having positive responses to any of the categories: evidence of severe complications, intervention-based criteria, organ system dysfunction, or investigation-based criteria. Thirteen percent of babies recruited did not meet any of the neonatal near-miss criteria (*N* = 53, 13.5%), despite having been identified as experiencing some type of complication or being admitted to the NICU.

**Fig. 1:**
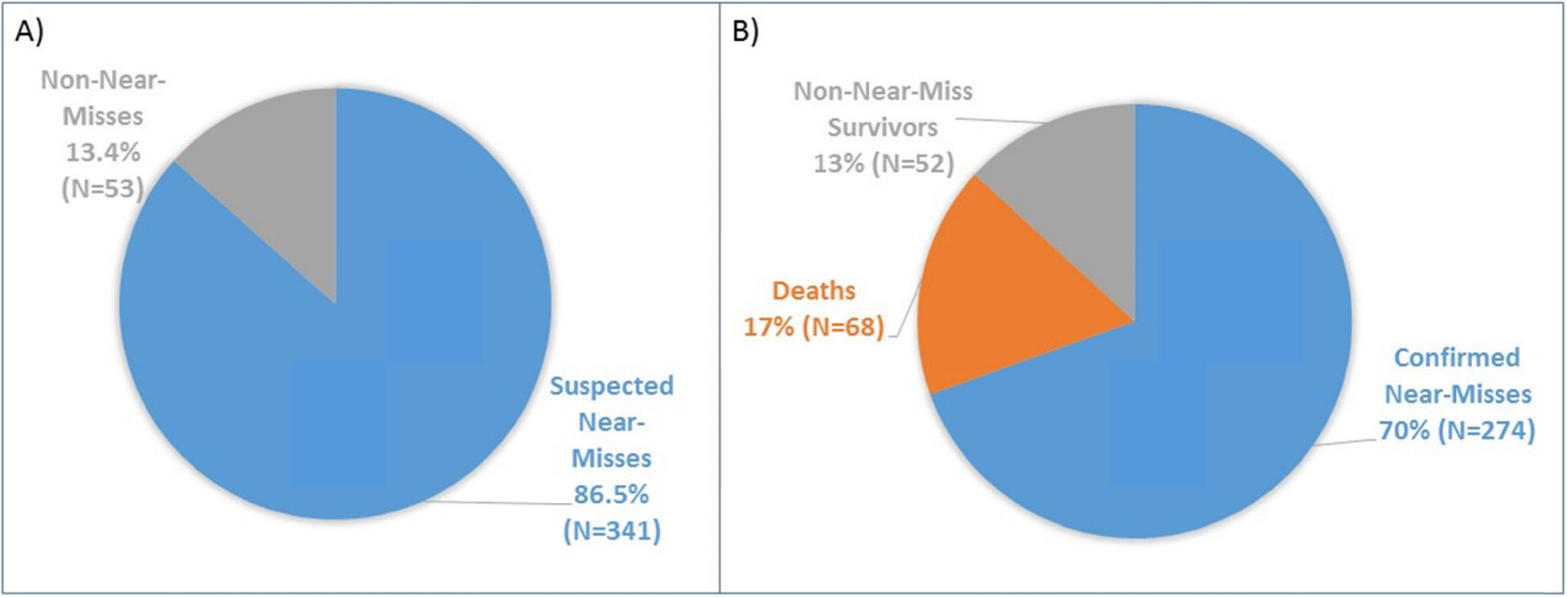
Participants at baseline (**a**) and at 28-day follow-up (**b**), *N* = 394

Figure 1 illustrates the composition of the sample at baseline (Fig. 1a) and at 28-day follow-up (Fig. 1b). At baseline, 341 newborns were classified as suspected near-misses, and 274 survived to 28 days to be classified as a confirmed near miss (80.4%). Sixty-eight newborns died between enrollment and 28-day follow-up. One out of the 53 babies not classified as a suspected near-miss (1.9%) died before 28 days. Sixty-seven of the babies classified as a suspected near-miss (19.6%) died before 28 days. When looked at as an odds ratio, being considered a suspected near-miss at enrollment according to the NNMAT conferred an 12.7-fold increased odds of death within 28 days when compared to not being considered a suspected ‘near-miss’. (OR: 12.7, *p* = .013, 95% CI 1.7–93.6).

The sensitivity of the NNMAT – or the proportion of newborns who died before 28 days who screened positively on NNMAT – was 98.5%. The specificity of the NNMAT – or the proportion of newborns who did not die by 28 days who had a negative result on the NNMAT – was 15.9%. The positive predictive value (PPV) of the NNMAT – the likelihood that being labeled as a confirmed near-miss predicts death among this sample of newborns – was 19.6%. The negative predictive value (NPV) – the likelihood that not being labeled as a confirmed near-miss would be associated with not dying – was 98.1%.

Table 3 illustrates the neonatal near-miss incidence and incidence ratios across the study sites, as well as overall. Overall, we found 57.7 neonatal near-misses per 1000 live births, with 36.2 per 1000 live births at KATH and 125.3 per 1000 live births at CCTH. Table 3 also illustrates institutional neonatal mortality rates, which overall stood at 105.6 neonatal deaths per 1000 live births for the four months of our study. Note that neonatal mortality also ranged from 53.9 per 1000 live births at CCTH to 122.1 per 1000 live births at KATH. While institutional neonatal mortality rates are limited to deaths that occur before a newborn is discharged (thus including newborns who die before being admitted to the NICU and excluding newborns who leave the hospital and die at home), these figures allowed for the calculation of the near-miss to mortality ratio, which suggested an overall ratio of 0.55 to 1. This differed by institution, with Cape Coast reporting 2.3 neonatal near-misses for every death, and KATH reporting approximately 3 deaths for every neonatal near-miss. (See Table 3).

**Table 3:** Neonatal Near-Miss Incidence and Incidence Rate at two Tertiary hospitals in Southern Ghana

	University of Cape Coast	Komfo Anokye Teaching Hospital	OVERALL
Live Births (April – July 2015)	1149	3596	4745
Institutionally-recorded Neonatal Deaths	62	439	501
Confirmed Neonatal Near-Misses (survived to 28 days)	144	130	274
Neonatal Near-Miss Incidence Rate per 1000 live births (95%CI)	125.3 (104.9, 145.8)	36.2 (29.9, 42.4)	57.7 (50.5, 65.01)
Neonatal Mortality Incidence Ratio per 1000 live births (95% CI)	53.9 (40.5, 67.4)	122.1 (110.7, 133.5)	105.6 (94.9, 116.2)
Near-Miss: Mortality Ratio	2.3: 1.0	0.30: 1.0	0.55: 1.0

Table 4 illustrates the results of multivariate logistic regression using mortality within the first 28 days as the outcome measure to determine which components of the NNMAT (indicated at enrollment) were most predictive of mortality. Model 1 shows the predictive value of the four overarching categories (evidence of severe complications, intervention-based criteria, organ system dysfunction, and investigation-based criteria). Model 1 suggests that evidence of severe complications and organ system dysfunction upon admission are the strongest predictors of neonatal mortality. Neither interventions nor investigations were predictive. Models 2, 3, 4, and 5 investigate the sub-components of each broader category, and Model 6 carries forward the individual items significant in Models 2, 3, 4, and 5 to create a composite model. Model 7 retains only those factors significant in Model 6. Model 7 shows that gestational age < 33 weeks confers a significantly increased odds of death (odds ratio: 3.0, *p* = 0.001), as does neurologic dysfunction (odds ratio: 2.3, *p* = 0.008), respiratory dysfunction (odds ratio 4.6, *p* <  0.001), and hemoglobin < 10 gd/dl (odds ratio: 6.4, *p* = 0.004). Performance of chest compressions was also included in the model, suggesting a seven times higher odds of death among babies who underwent chest compressions, yet the *p*-value of 0.019 exceeded our threshold of 0.01.

**Table 4:** Multivariate Logistic Regression Models Predicting Odds of Neonatal Mortality within 28 days, Based on Assessment at Enrollment

Variable Name	Model 1 (Major Near-Miss Categories) Pseudo R2 = 0.0982			Model 2 (Aspects of Severe Complications) Pseudo R2 = 0.0746			Model 3 (Aspects of Interventions) Pseudo R2 = 0.0982			Model 4 (Aspects of Organ Dysfunction) Pseudo R2 = 0.1517			Model 5 (Aspects of Investigations) Psuedo R2 = 0.0133			Model 6 (Model with key predictors from previous models) Pseudo R2 = 0.2176			Model 7 (Final Model) Pseudo R2 = 0.2092		
	OR	*P* value	95% CI	OR	*P* value	95% CI	OR	*P* value	95% CI	OR	*P* value	95% CI	OR	*P* value	95% CI	OR	*P* value	95% CI	OR	*P* value	95% CI
Severe Complications	2.1	0.041*	1.0–4.1																		
Apgar Score < 7 at 5 min				1.3	0.396	0.7–2.3															
Gestational Age < 33 wks				6.4	< 0.001*	2.2–18.2										3.1	0.001*	1.6–6.0	3.0	0.001*	1.6–5.9
Birthweight < 1800 g				0.5	0.194	0.2–1.4															
Suspected subgaleal bleed				1.3	0.576	0.5–3.5															
Major congenital abnormality				2.5	0.078	0.9–6.9															
Axillary temperature < 35 or > 39				1.9	0.023	1.1–3.6										1.7	0.09	0.9–3.3			
Severe jaundice that required blood exchange				2.2	0.265	0.6–8.4															
Surgery in the first week				1.0	1.0	0.9–10.7															
Interventions	1.3	0.555	0.5–3.1																		
Resuscitation at delivery							0.9	0.737	0.4–1.8												
Resuscitation in NICU							2.0	0.069	0.9–4.1												
Chest compressions							9.6	0.007*	1.8–50.3							8.3	0.013*	1.6–44.4	7.2	0.019	1.4–37.5
IV fluid bolus							1.3	0.426	0.7–2.6												
Any intubation							0.7	0.748	0.1–6.4												
Double phototherapy							1.1	0.932	0.3–3.2												
Double volume exchange blood transfusion							1.1	0.943	0.2–4.5												
Oxygen therapy							2.0	0.018*	1.1–3.7							0.8	0.468	0.4–1.6			
Caffeine citrate aminophylline							0.9	0.856	0.4–2.2												
Thermal protection							1.4	0.379	0.7–3.0												
Organ System Dysfunction	3.8	< 0.001*	1.9–7.2																		
Cardiovascular										2.1	0.174	0.7–6.4									
Neurologic										2.9	< 0.001*	1.6–5.2				2.2	0.010*	1.2–4.3	2.3	0.008*	1.2–4.2
Respiratory										4.5	< 0.001*	2.4–8.3				5.0	< 0.001*	2.4–10.1	4.6	< 0.001*	2.4–8.8
Renal										1	(omitted)	–				3.0	0.152	0.6–13.8			
Gastroenterologic										0.4	0.435	0.04–4.2									
Investigations	1.6	0.327	0.6–4.2																		
Hematocrit < 30% in first week													0.7	0.774	0.1–5.8						
Hemoglobin <10 gd/dl													4.3	0.044*	1.0–18 .4	6.9	0.003*	1.9–24.4	6.4	0.004*	1.8–22.8
Blood culture done													0.9	0.649	0.5–1.6						
Blood culture positive													3.3	0.194	0.5–20.4						

*Significant at *p* < 0.05

## Discussion

This study showed that out of 394 newborns with complications, 341 (86.5%) were initially classified as suspected neonatal near-misses using the NNMAT, and 274 (70%) were ultimately classified as confirmed near-misses as they survived to 28 days. Those newborns with complications who were classified as suspected near-misses at enrollment using the NNMAT had 12 times the odds of dying before 28 days than those not classified as suspected near-misses. While most newborns qualified as suspected and confirmed near-misses via intervention-based criteria, nearly two-thirds of newborns qualified as suspected and confirmed near-misses based on two or more of the four NNMAT categories. When disaggregated, the most predictive elements of the NNMAT were gestational age < 33 weeks, neurologic dysfunction, respiratory dysfunction and hemoglobin < 10 gd/dl. The overall ratio of near-misses to deaths was 0.55:1, yet this varied across the study sites.

This research suggests that the NNMAT is an effective tool for assessing neonatal near-misses in low-resource settings. Similar to the findings of Kale et al., [3] the NNMAT indicates that pragmatic criteria (which we termed ‘evidence of severe complications’) are useful, given that 66% of newborns were classified as neonatal near-misses based on these criteria. However, as our findings and the recommendations of Kate et al. indicate, relying solely upon pragmatic markers such as Apgar scores, gestational age and birthweight is likely to underestimate the true burden of neonatal near misses. Using the NNMAT we identified a total of 274 confirmed neonatal near-misses from a sample of 394 babies with complications, more than a quarter of which would not have been classified as a near miss if we only utilized pragmatic markers. Our results also indicate that the majority of newborns classified as both suspected and confirmed near misses were classified based on more than one NNMAT category, with only 69 of the 274 confirmed near-misses classified as such based on a single NNMAT category. This argues against oversimplifying neonatal near-miss assessment, instead suggesting the need for a balance between a comprehensive assessment and tools that are still applicable in low-resource settings with limited access to technologically advanced assessments and interventions.

The NNMAT’s sensitivity was 98.5%, indicating a high likelihood of identifying babies at risk of death before 28 days. However, the NNMAT had a positive predictive value of 19.6%, which could be considered relatively low in its ability to successfully predict death. However, the NNMAT is designed to identify those babies who almost died but ultimately survived, and in that case, a low PPV is less worrisome than it might be in other contexts. Interestingly, the NNMAT’s negative predictive value was extremely high (98.1%), indicating that those not classified as a near miss are extremely unlikely to die before 28 days. It is also possible that the NNMAT may be most effective and efficient if directed to a high risk population, such as those newborns admitted to the NICU rather than including all newborns with complications.

This study is an important addition to the neonatal near-miss literature. We leveraged what was known about maternal near-miss assessment in LMICs and combined that with extant learnings from the nascent field of neonatal near-miss research to generate and test a tool across two tertiary care centers in Ghana. Our findings build on the results of Nakimuli et al. [10] in Uganda who focused on neonatal near-misses attributable to severe obstetric complications and Ronsmans et al., [11] who identified neonatal near-misses based on organ dysfunction markers in hospitals in Benin, Burkina Faso, and Morocco. As in our study, Ronsmans et al. found that many neonatal near-misses occurred among babies who were not considered premature, low birthweight, or with a low 5-min Apgar score, [11] which raises questions about the quality of birth and early postnatal care being offered. Unfortunately, direct comparisons of neonatal near-miss rates across studies are not possible due to the wide variability in how the definition of neonatal near-miss is operationalized.

This study has several important implications. First, the NNMAT illustrates that it is possible to use a simple tool to assess markers that are readily observed in low-resource settings to categorize newborns with complications as suspected neonatal near-misses or not, and then follow them to 28 days to confirm whether they were indeed a near-miss. While some might argue that the pragmatic markers of gestational age, birth weight, and Apgar score used in previous research are sufficient for that purpose, our results indicate that evidence of respiratory distress, neurologic dysfunction and low hemoglobin levels provide valuable additional information with which to predict outcomes. A newborn’s positive classification as a suspected near-miss on the NNMAT conferred a statistically significant, 12-fold increased risk of death before 28 days. This has enormous implications for clinical management, including the possibility of implementing a standard methodology for assessing newborns and alerting staff to especially high-risk babies that require additional attention. Given that most babies admitted to a NICU are seriously ill, it may be difficult to imagine further triage. Yet the NNMAT indicates that further triage may indeed be warranted, especially among more junior-level providers who may not immediately recognize the seriousness of newborn symptoms and are likely to be the ones in charge during overnight and weekend shifts. Such a precedent is illustrated by the British Association of Perinatal Medicine’s new guidelines for management of prematurity, including risk stratification of premature newborns based on some of the same factors identified in the NNMAT: birthweight, gender, multiple births, and congenital anomalies [19]*.* In addition, these findings also suggest a role for programs that follow newborns beyond their discharge from the NICU. Current global movements in newborn care are emphasizing more than survival – including an effort to ensure that newborns don’t just survive, but that they have every opportunity to thrive [20]*.*

Our findings also suggest that the NNMAT may be useful in driving the development of quality improvement programs, as individual facilities identify the most common challenges they face through routine monitoring of NNMAT scores against mortality outcomes. Potential differences across individual facilities raises another important implication of the NNMAT. We found wide variability in near-miss to mortality ratios across tertiary care hospitals that we assumed would be similar, suggesting that there may be case-mix issues that preclude ‘fair’ comparisons of outcomes across settings. In other words, the NNMAT may provide a mechanism for individual facilities to assign scores to patients entering the NICU that can then be used to contextualize subsequent outcomes data.

This study has several limitations worth noting. First, our initial sample size of 441 was reduced to 394 we were able to follow to 28 days post-birth. This loss-to-follow-up was more pronounced in the non-near-miss group, potentially affecting our findings. A second limitation is that we used aggregate institutional neonatal mortality rates as the comparison against observed rates of neonatal near-misses to create the ratio of near-misses to deaths. Institutional neonatal mortality rates typically include any baby who dies in the facility, including those born outside the facility (which may inflate the numbers), but they do not include those babies who die after discharge (which may underestimate the mortality burden). These rates also use live births in the facility as their denominator, while potentially including babies born outside their facilities in the numerator. Nonetheless, this imperfectly calculated rate allows us to compare neonatal near-misses (which in this study included those born outside the facility, and did not include data on what happens to newborns after discharge) with institutional neonatal mortality, providing an approximation of the ratio of near-misses to deaths. While these calculations were consistent across facilities, and are thus comparable, the ratios of neonatal near-misses to neonatal mortality presented here must not be confused with population-level neonatal mortality rates. Another limitation of our study relates to the use of yes/no/don’t know in the NNMAT. In settings where record keeping or communication between providers is poor, or where certain interventions or investigations are not available, or where laboratory tests take a long time to come back, it is possible that the “don’t know” responses could lead to an underestimation of the number of babies who might qualify as a potential near-miss. However, we believe the NNMAT is broad and inclusive enough to capture other risk markers even if some are not known. The timing of the near-miss assessments is also worthy of discussion. We recruited participants as soon as possible after admission, and as such our NNMAT assessments were typically conducted in the first day or two after birth. Assessments completed later in the first week might have yielded different exposures to treatments or interventions and thus our findings may look different. Given lack of clarity regarding the ideal time to assess a newborn for near-miss status, future research is warranted that explores ideal timing of the NNMAT administration. Finally, our study is limited in its duration of follow-up. Future research that follows newborns to 3, 6, 9 and 12 months is warranted to determine the long term impact of neonatal near-miss events.

## Conclusions

In summary, we have developed, evaluated, and demonstrated the successful use of a pragmatic heuristic in the form of the NNMAT to identify neonatal near misses. We believe this approach has significant systems-level, continuous quality improvement, clinical and policy-level implications.

## Data Availability

The datasets used and/or analysed during the current study are available from the corresponding author on reasonable request.

## Abbreviations

CCTH: Cape Coast Teaching Hospital
KATH: Komfo Anokye Teaching Hospital
KBTH: Korle Bu Teaching Hospital
NICU: Neonatal Intensive Care Unit
NNM: Neonatal mortality
NNMAT: Neonatal Near-Miss Assessment Tool
OR: Odds Ratio
WHO: World Health Organization
